# Streptococcus constellatus Empyema: A Case Highlighting the Need for Timely Surgical Intervention

**DOI:** 10.7759/cureus.72199

**Published:** 2024-10-23

**Authors:** Supriya Peshin, Mishell Siles Borda, Nirmay Sonar, Vaishnavi Kulkarni, Asfand Khattak

**Affiliations:** 1 Internal Medicine, Norton Community Hospital, Norton, USA; 2 Medical Education, MGM (Mahatma Gandhi Mission) Institute of Health Sciences, Navi Mumbai, IND

**Keywords:** dornase alfa, pleural empyema, streptococcus constellatus, tissue plasminogen activator (tpa), video-assisted thoracoscopic surgery (vats)

## Abstract

This case report explores the intricate challenges of diagnosing and managing empyema caused by *Streptococcus constellatus*, particularly in patients with predisposing factors such as alcohol abuse and underlying respiratory conditions. We present a 34-year-old male patient with a medical history of hypertension, peripheral neuropathy, and alcohol abuse who developed empyema. Despite an initial presentation at another facility with symptoms mimicking a myocardial infarction and unremarkable chest X-ray results, his condition worsened, leading to a subsequent emergency department visit. The patient's persistent pleuritic chest pain and fever were initially managed with ibuprofen and steroids, which proved ineffective. Upon re-evaluation, he exhibited hemodynamic instability, and imaging revealed a moderate pleural effusion. An urgent chest tube placement drained over 1600 ml of purulent fluid, with cultures confirming *S. constellatus*. The patient was treated with broad-spectrum antibiotics and intrapleural administration of tissue plasminogen activator and dornase alfa; however, unresolved effusion necessitated video-assisted thoracoscopic surgery (VATS). This intervention successfully eradicated the infection. The case underscores the importance of considering less common pathogens like *S. constellatus* in atypical empyema cases and emphasizes the critical role of VATS in resolving complex pleural infections. Early recognition, comprehensive management strategies, and a high index of clinical suspicion are vital to reducing morbidity and mortality associated with such infections.

## Introduction

Empyema, characterized by the accumulation of pus in the pleural space, can complicate pneumonia or arise from various infections, leading to significant morbidity and mortality if not promptly addressed [1]. The administration of empiric broad-spectrum antibiotics, including coverage for anaerobic organisms, is essential in the initial management of empyema, ensuring comprehensive treatment while awaiting specific pathogen identification [2]. *Streptococcus constellatus*, a member of the *Streptococcus milleri* group, is particularly known for causing abscesses and invasive infections such as empyema, especially in individuals with predisposing factors like alcohol abuse and underlying respiratory conditions [3]. This case report presents the clinical challenges in diagnosing and managing empyema in a 34-year-old male patient with a history of hypertension, peripheral neuropathy, and alcohol abuse, who developed the condition due to *S. constellatus*.

## Case presentation

A 34-year-old man with a past medical history of hypertension, peripheral neuropathy, and alcohol abuse presented to the emergency department (ED) with a chief complaint of severe worsening pleuritic chest pain for two to three weeks before presentation. Initially, the patient had presented to another facility, suspecting a myocardial infarction. A chest X-ray was obtained at that time and was unremarkable. The patient was discharged with ibuprofen for pain management. However, the chest pain persisted and worsened with deep inspiration and coughing. Follow-up with his primary care physician led to a prescription of steroids, which did not alleviate the symptoms. On the morning of the ED presentation, the patient reported a fever of 100.7°F. He acknowledged a remote history of alcohol abuse but reported current social drinking, denying binge drinking or sick contacts. 

In the ED, the patient was hemodynamically unstable with a heart rate in the 130s, respiratory rate of 25, and oxygen saturation of 95% on 5L per minute oxygen via nasal cannula. Laboratory studies revealed a white blood cell count of 17.4 x 10^9^/L and a lactate level of 2 mmol/L. An arterial blood gas (ABG) indicated hypoxia (Table 1).

**Table 1:** Summary of laboratory investigations on admission

Test	Results	Reference Range	Interpretation
White Blood Cell Count	17.4 x 10^9^/L	4.0 - 11.0 x 10^9^/L	Elevated (indicative of infection)
Lactate Level	2 mmol/L	0.5 - 1 mmol/L	Elevated (suggestive of sepsis)
Arterial Blood Gas	Hypoxia	pO₂ > 75 mmHg	Reduced (suggestive of respiratory failure)
Fever	100.7°F	< 98.6°F	Febrile (indicative of infection)
Oxygen Saturation	95% on 5L O₂	> 95%	Hypoxia requiring oxygen therapy

**Figure 1:**
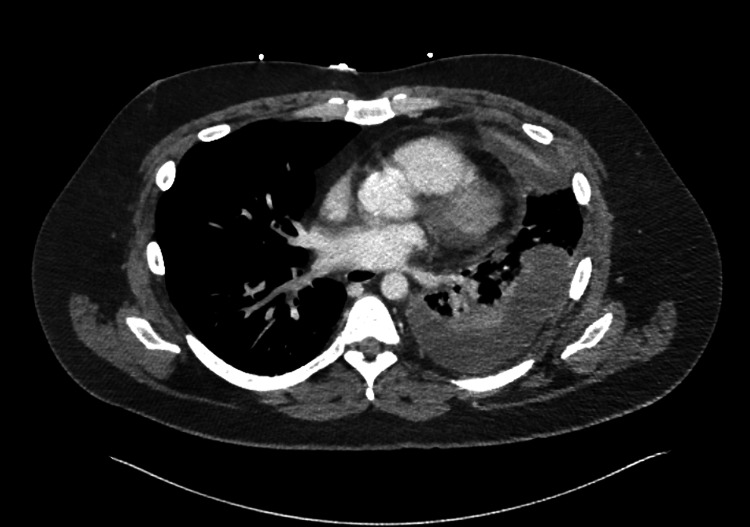
CT scan showing large left pleural effusion/empyema before chest tube placement

**Figure 2:**
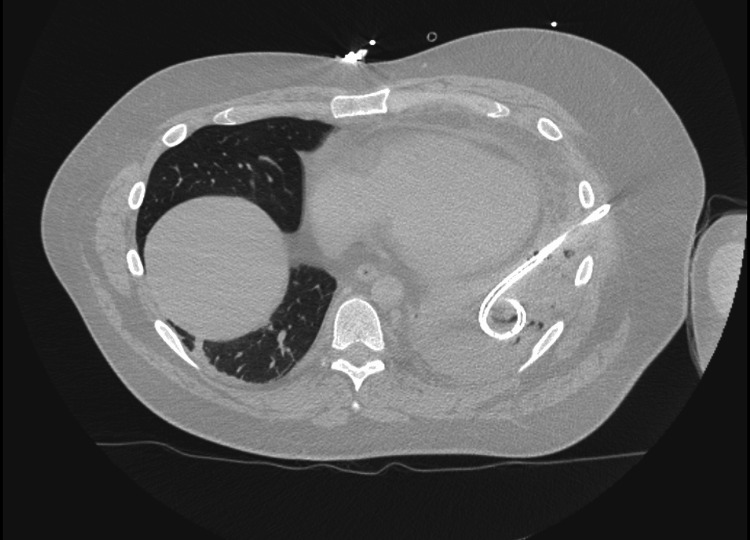
CT scan post chest tube placement

Imaging studies, including a chest X-ray and CT angiogram, demonstrated a moderate left pleural effusion and atelectasis of the majority of the left lower lobe (Figure 1). The pulmonary team was consulted for urgent chest tube placement, which initially drained over 600 ml of purulent fluid, brown and cloudy in color. The chest tube continued to drain over 1000 ml of fluid spontaneously (Figure 2).

Pleural fluid analysis confirmed exudative fluid, and cultures grew *S. constellatus*. The patient was empirically started on cefepime, vancomycin, and metronidazole, which were later de-escalated based on culture results and clinical improvement. The patient also received chest tube infusions of tissue plasminogen activator (tPA) and dornase alfa to facilitate further fluid drainage. Despite these measures, repeated imaging continued to show unresolved pleural effusion. Cardiothoracic surgery was consulted for video-assisted thoracoscopic surgery (VATS), which successfully eradicated the infected effusion (Table 2).

**Table 2:** Comparison of chest imaging results

Imaging Modality	Findings	Interpretation
Chest X-ray (Initial)	Unremarkable	No significant findings
Chest CT (Pre-chest tube)	Moderate left pleural effusion, left lower lobe atelectasis	Suggestive of empyema
Chest CT (Post-chest tube)	Drained pleural effusion, residual effusion noted	Effective drainage with residual fluid

## Discussion

Empyema, a collection of pus in the pleural space, can complicate pneumonia or arise from various infections, and is associated with significant morbidity and mortality if not promptly recognized and managed [1]. This case report highlights the challenges in diagnosing and managing a patient with hypertension, peripheral neuropathy, and alcohol abuse, who developed empyema due to *S. constellatus*.

Empiric broad-spectrum antibiotics, including coverage for anaerobic organisms, are essential until specific pathogens are identified [2]. *S. constellatus*, a member of the *S. milleri *group, is known for causing abscesses and invasive infections, including empyema, particularly in individuals with risk factors like alcohol abuse and underlying respiratory conditions [3]. The initial unremarkable chest X-ray in this case emphasizes the importance of considering empyema in patients with persistent pleuritic chest pain and respiratory symptoms, even when initial imaging is inconclusive [4]. The patient's alcohol abuse may have increased his susceptibility to invasive infections by impairing immune function and respiratory health [5].

Although *S. constellatus* is an uncommon cause of empyema, it is significant in patients with predisposing factors. Prompt recognition, appropriate antimicrobial therapy, and timely surgical intervention are crucial for successful outcomes [6]. In complex effusions, intrapleural administration of tissue plasminogen activator and dornase alfa can enhance drainage, though surgical intervention such as VATS may be necessary for complete resolution [7].

## Conclusions

This case highlights the complexities and challenges in diagnosing and managing empyema caused by *S. constellatus*, especially in patients with risk factors such as alcohol abuse and underlying respiratory conditions. Despite initial diagnostic difficulties, the patient's condition was successfully managed through a combination of timely surgical intervention and targeted antibiotic therapy. The case underscores the critical need for high clinical suspicion and comprehensive management strategies when encountering similar presentations, as early intervention can significantly reduce morbidity and mortality associated with empyema. It also emphasizes the importance of considering less common pathogens like *S. constellatus* in atypical cases of empyema and the role of VATS in resolving complicated pleural infections when conventional medical management is insufficient.

## References

[1] Bobbio A, Bouam S, Frenkiel J (2021). Epidemiology and prognostic factors of pleural empyema. Thorax.

[2] Reigadas E, Alcalá L, Marín M, Muñoz-Pacheco P, Catalán P, Martin A, Bouza E (2016). Clinical significance of direct cytotoxicity and toxigenic culture in Clostridium difficile infection. Anaerobe.

[3] Porta G, Rodríguez-Carballeira M, Gómez L, Salavert M, Freixas N, Xercavins M, Garau J (1998). Thoracic infection caused by Streptococcus milleri. Eur Respir J.

[4] Davies HE, Davies RJ, Davies CW (2010). Management of pleural infection in adults: British Thoracic Society pleural disease guideline 2010. Thorax.

[5] Simou E, Leonardi-Bee J, Britton J (2018). The effect of alcohol consumption on the risk of ARDS: a systematic review and meta-analysis. Chest.

[6] Finley C, Clifton J, Fitzgerald JM, Yee J (2008). Empyema: an increasing concern in Canada. Can Respir J.

[7] Rahman NM, Maskell NA, West A (2011). Intrapleural use of tissue plasminogen activator and DNase in pleural infection. N Engl J Med.

